# Navigating the Challenges of Takayasu Arteritis in a 26-Year-Old Female With Recurrent Chest Pain and a Complex Medical History

**DOI:** 10.7759/cureus.48569

**Published:** 2023-11-09

**Authors:** Tomas Escobar Gil, Alexandra C Millhuff, Satya A Gbadamosi Muhammad, Lucas K Akes, Saumya Joshi, Aaron J Jones

**Affiliations:** 1 Internal Medicine, University of New Mexico School of Medicine, Albuquerque, USA; 2 Internal Medicine, University of New Mexico Hospital, Albuquerque, USA; 3 Internal Medicine, Civil Hospital Ahmedabad, Ahmedabad, IND

**Keywords:** autoimmune, large vessel vasculitis, aortitis, radiology, vasculitis, rheumatology, takayasu arteritits

## Abstract

Takayasu arteritis, a rare and complex vasculitis, presents unique diagnostic and management challenges, particularly when encountered in young adults. We present the case of a 26-year-old female with obesity, prediabetes, hepatic steatosis, an adnexal cyst, *Helicobacter pylori* gastritis, and asthma, who was transferred to our facility due to concerns about aortitis. Her presentation to the referring institution included dysphagia, heartburn that responded to over-the-counter antacids, and recurrent episodes of stabbing chest pain, which had been occurring intermittently since the age of 17.

Previous visits to the emergency room for these symptoms had been approached as gastritis, the last being two weeks before this episode. On evaluation, laboratory findings revealed elevated inflammatory markers, and subsequent imaging studies identified extensive circumferential wall thickening of the ascending thoracic aorta, suggestive of aortitis, and the patient was transferred to our institution. The patient’s complex medical history and psychosocial stressors, including estrangement from her family, added to the intricacies of her case.

Rheumatology consultation was instrumental in guiding further evaluation and management. A diagnosis of Takayasu arteritis with large vessel vasculitis was considered, supported by positron emission tomography-computed tomography findings showing significant metabolic activity in major arteries. The patient was initiated on prednisone therapy, *Pneumocystis jirovecii* pneumonia prophylaxis, and methotrexate. Ongoing monitoring for disease activity and medication side effects was emphasized.

This case highlights the importance of considering rare conditions such as Takayasu arteritis in young adults with atypical presentations and underscores the need for comprehensive, multidisciplinary care that addresses not only the medical aspects but also the psychosocial well-being of the patient.

## Introduction

Aortitis is a broad term that denotes inflammation of the aortic wall [1]. Presenting symptoms tend to be nonspecific, such as chest pain, back pain, abdominal pain, fatigue, and headache. Furthermore, it is often challenging to interpret the data around aortitis because there has been no consensus that explains all forms of etiology, nor is there a generally accepted classification [2,3]. Our case focuses on Takayasu aortitis (TA), a less common subtype of aortitis that primarily affects the abdominal and thoracic aorta.

This case report seeks to shed light on the challenges associated with TA and contribute to raising awareness about this condition. By sharing the complexities involved in diagnosing and managing this condition, we aim to enhance the understanding of healthcare providers and encourage early recognition of TA. In the subsequent sections, we present the case of a 26-year-old female with a complex medical history, recurrent chest pain, and other systemic symptoms, illustrating the nuances of diagnosing and addressing TA.

## Case presentation

A 26-year-old female patient of Hispanic race presented with a complex medical history and a chief complaint of recurrent, severe chest pain. These episodes had been recurring intermittently since the age of 17, characterized by sharp and stabbing pain with an intensity rating of 7/10, each lasting several hours. They were also associated with heartburn and responded to antacids. Notably, she had recently experienced two episodes within two weeks, more frequent than her typical intermittent presentations.

Her past medical history included obesity, prediabetes, hepatic steatosis, an adnexal cyst, and asthma, with a family history of diabetes. Upon admission, comprehensive laboratory tests revealed significant findings, including an elevated white blood cell count of 12.8 × 10^9^/L, an increased C-reactive protein (CRP) of 1.3 mg/dL, and an elevated erythrocyte sedimentation rate (ESR) of 79 mm/hour. Additionally, her liver function tests showed elevated alanine aminotransferase of 163 U/L.

**Figure 1:**
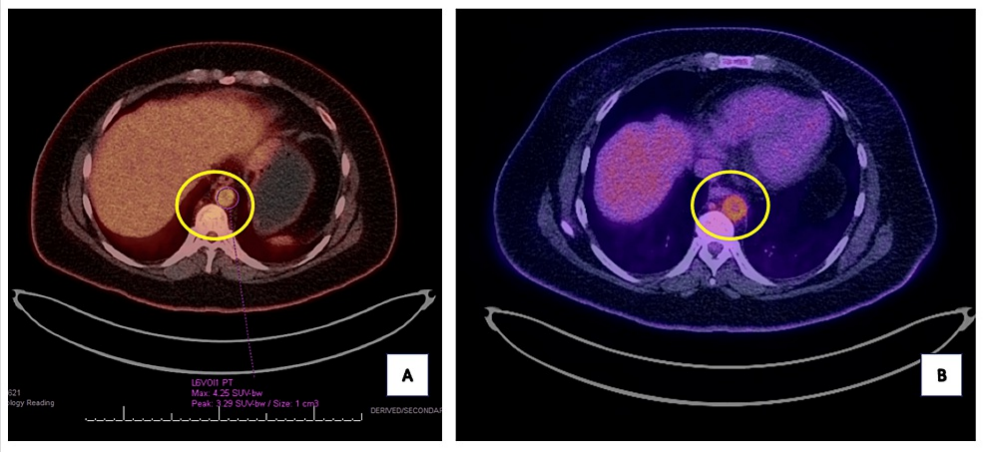
Positron emission tomography CT scan of the abdomen and pelvis. The yellow circles in A and B show significant metabolic activity in the descending aorta to subclavian arteries, iliofemoral, and popliteal arteries, indicating active vasculitis.

**Figure 2:**
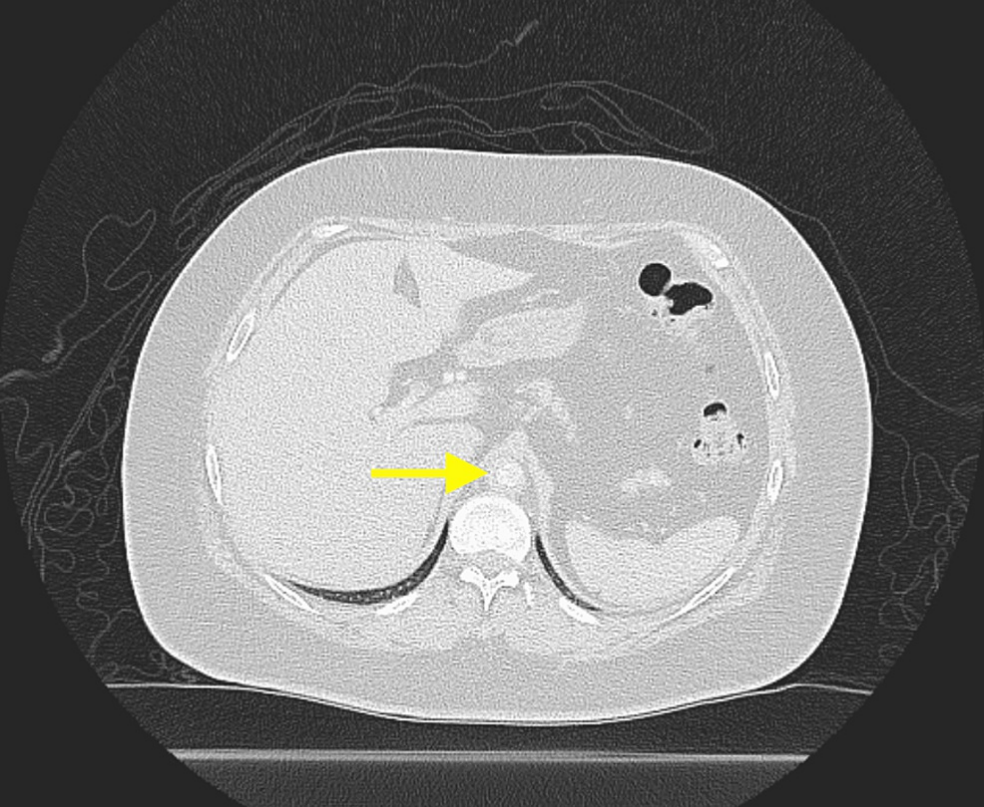
CT imaging of the abdomen and pelvis with contrast. The yellow arrow is pointing toward an extensive circumferential wall thickening along the ascending thoracic aorta, suggesting an inflammatory vasculitis, likely Takayasu arteritis.

Imaging studies were crucial in shaping the diagnostic process. A positron emission tomography (PET) CT scan (Figures 1A, 1B) revealed significant metabolic activity in the descending aorta to subclavian arteries, iliofemoral, and popliteal arteries, indicating active vasculitis. Notably, no intracranial abnormalities were detected. Additionally, a CT of the abdomen and pelvis with contrast (Figure 2) demonstrated extensive wall thickening along the descending thoracic aorta and suprarenal abdominal aorta with mild narrowing of the aortic lumen, as well as a 3.6 cm × 2.6 cm left ovarian cyst.

Given the complexity of the case and the need for specialized evaluation, Vascular Surgery and Rheumatology consultations were requested. Vascular Surgery did not recommend any surgical interventions. Rheumatology, on the other hand, recommended prednisone therapy at a dose of 1 mg/kg (equivalent to 80 mg daily) as treatment for vasculitis. Prophylaxis for *Pneumocystis jirovecii* pneumonia with trimethoprim/sulfamethoxazole was also administered while the patient was on high-dose steroids. Additionally, methotrexate (MTX) was introduced, with a weekly oral dose of 20 mg, along with daily folic acid supplementation to mitigate potential side effects.

Contraception counseling was provided due to the patient’s use of an etonogestrel implant, and her psychosocial stressors, including estrangement from her family and the demands of work, school, and pottery classes, were taken into consideration during her care, emphasizing the importance of holistic management.

The patient was discharged with a comprehensive care plan in place, highlighting the significance of ongoing monitoring. Regular laboratory tests, encompassing complete blood counts, comprehensive metabolic panels, ESR, and CRP, were scheduled every three months for both disease activity and MTX monitoring. Follow-up appointments with the Rheumatology team were planned to monitor treatment response and assess disease progression.

## Discussion

Giant cell arteritis (GCA) and TA are the two forms of large vessel vasculitides, and both are known to affect the aorta and its major branches [4]. GCA primarily affects the extracranial branches of the carotid, while TA, less frequently encountered, predominantly involves the thoracic and abdominal aorta and its branches [4]. Our case focuses on TA.

TA is a rare form of large vessel vasculitis primarily affecting the aorta and its major branches [5]. This condition predominantly affects young to middle-aged women, as exemplified by the case of the 26-year-old female patient in this report [2]. Asian women have also been reported to be more commonly affected [4]. Contemporary incidence estimates for TA in the United States are lacking, but it has been estimated to affect 2.6 new cases per million persons per year [6]. The mean age at diagnosis is between 25 and 30 years, and women have been reported to comprise 75%-97% of cases [6].

The pathogenesis of TA remains incompletely understood, but it is considered an autoimmune condition characterized by granulomatous inflammation of the vessel wall [7]. TA symptoms can resemble those of atherosclerotic cardiovascular disease, and patients often complain of recurrent episodes of chest pain. If patients persistently consult for similar symptoms despite adherence to prescribed medications for coronary artery disease, especially in young female patients, it should raise red flags and prompt further evaluation [8]. The main distinguishing feature between GCA and TA is age, with TA usually affecting patients younger than those with GCA [4]. TA often lacks specific markers and is frequently underrecognized by clinicians [9]. Because there is no standardization of terminology, and no specific histological features separating Takayasu’s aortitis from non-specific granulomatous aortitis, the approach to treatment is variable [10]. In the presented case, the patient exhibited a complex clinical course marked by recurrent, severe chest pain and a history of multiple episodes of similar symptoms.

The diagnosis of TA requires a high index of suspicion and a multifaceted approach, relying heavily on a combination of clinical evaluation, laboratory findings, and imaging modalities [5,11]. Angiography, including CT angiography and magnetic resonance angiography, plays a central role in identifying characteristic features such as arterial stenosis, occlusion, and vascular wall thickening, providing insights into vascular inflammation and damage [3,12]. In our case, a PET-CT was done with equal efficacy in detecting the vasculitis. However, analyzing retrospectively, the latter imaging modalities should have been considered with this diagnosis at hand. There is rarely a place for a biopsy due to the risks of performing invasive procedures in an already inflamed aorta [4]. Moreover, elevations in inflammatory markers such as ESR and CRP contribute to the diagnostic process, indicating ongoing inflammation [13].

While clinical symptoms can vary, imaging studies are pivotal in establishing the diagnosis. The CT angiogram, which revealed extensive circumferential wall thickening along the ascending thoracic aorta, played a key role in diagnosing this patient’s TA. Such findings, in conjunction with characteristic angiographic abnormalities and a comprehensive radiologic approach, contribute to the diagnosis, aligning with the diagnostic criteria for TA [3].

Treatment of TA primarily revolves around suppressing the inflammatory process with glucocorticoids and immunosuppressive agents [8]. Prednisone is commonly used as an initial therapy to control inflammation. Additionally, MTX, azathioprine, or mycophenolate mofetil may be added to enable steroid tapering and maintain disease remission. In some cases, biological agents, such as tumor necrosis factor inhibitors, are considered when conventional treatment fails. Regular monitoring of disease activity and medication side effects is essential in the long-term management of TA [6,7]. In the case discussed, the patient received prednisone as the initial therapy, followed by the introduction of MTX and folic acid to enable steroid tapering while controlling disease activity. This comprehensive approach to management aims to prevent further vascular damage and complications, making early diagnosis and treatment crucial in improving patient outcomes [14].

The prognosis of TA varies, and the long-term outcome can be influenced by several factors. Early diagnosis and prompt initiation of appropriate treatment are associated with better outcomes, including disease control and prevention of complications [14]. However, TA can lead to significant morbidity if left untreated or inadequately managed [6]. Vascular complications, such as arterial stenosis, occlusion, or aneurysms, can result in organ damage and dysfunction [6]. Regular monitoring of disease activity and potential relapses is crucial, as the disease course can be unpredictable [14]. With vigilant management and adherence to treatment, many TA patients can achieve and maintain remission, leading to an improved quality of life [8].

The psychosocial aspects of this patient’s life should not be overlooked. Stress and a lack of familial support may have contributed to her disease severity and exacerbation. Therefore, a holistic approach to care, considering the patient’s psychosocial needs, is of utmost importance in managing TA effectively.

## Conclusions

In summary, TA, a rare large vessel vasculitis, poses unique diagnostic and management challenges, particularly when it manifests atypically, as illustrated in this case report. It highlights the necessity for a multidisciplinary approach, including rheumatology expertise, to effectively diagnose and manage this condition. With no specific diagnostic markers, comprehensive imaging and thorough clinical evaluation are pivotal in confirming the diagnosis. Furthermore, this case underscores the importance of regular monitoring of disease activity in the long-term management of TA. Raising awareness and understanding of this rare condition among healthcare providers is vital to ensure timely and appropriate care, ultimately improving the outcomes and overall well-being of patients with TA.
